# Evolution of Structural and Optical Properties of ZnO Nanorods Grown on Vacuum Annealed Seed Crystallites

**DOI:** 10.3390/nano8020068

**Published:** 2018-01-26

**Authors:** Waqar Khan, Fasihullah Khan, Hafiz Muhammad Salman Ajmal, Noor Ul Huda, Ji Hyun Kim, Sam-Dong Kim

**Affiliations:** 1 Division of Electronics and Electrical Engineering, Dongguk University, Seoul 100-715, Korea; waqarkyz@gmail.com (W.K.); fasihullah.khan@dongguk.edu (F.K.); salman.gikian@gmail.com (H.M.S.A.); noor.ul.h694@gmail.com (N.U.H.); 2 School of Advanced Materials Science and Engineering, Sungkyunkwan University, Suwon-Si, Gyeonggi-Do 16419, Korea; poket350@gmail.com

**Keywords:** ZnO nanorods, vacuum annealing, photoluminescence, hydrothermal process, surface defects

## Abstract

In this study, the ambient condition for the as-coated seed layer (SL) annealing at 350 °C is varied from air or nitrogen to vacuum to examine the evolution of structural and optical properties of ZnO nanorods (NRs). The NR crystals of high surface density (~240 rods/μm^2^) and aspect ratio (~20.3) show greatly enhanced (002) degree of orientation and crystalline quality, when grown on the SLs annealed in vacuum, compared to those annealed in air or nitrogen ambient. This is due to the vacuum-annealed SL crystals of a highly preferred orientation toward (002) and large grain sizes. X-ray photoelectron spectroscopy also reveals that the highest O/Zn atomic ratio of 0.89 is obtained in the case of vacuum-annealed SL crystals, which is due to the effective desorption of hydroxyl groups and other contaminants adsorbed on the surface formed during aqueous solution-based growth process. Near band edge emission (ultra violet range of 360–400 nm) of the vacuum-annealed SLs is also enhanced by 44% and 33% as compared to those annealed in air and nitrogen ambient, respectively, in photoluminescence with significant suppression of visible light emission associated with deep level transition. Due to this improvement of SL optical crystalline quality, the NR crystals grown on the vacuum-annealed SLs produce ~3 times higher ultra violet emission intensity than the other samples. In summary, it is shown that the ZnO NRs preferentially grow along the wurtzite c-axis direction, thereby producing the high crystalline quality of nanostructures when they grow on the vacuum-annealed SLs of high crystalline quality with minimized impurities and excellent preferred orientation. The ZnO nanostructures of high crystalline quality achieved in this study can be utilized for a wide range of potential device applications such as laser diodes, light-emitting diodes, piezoelectric transducers and generators, gas sensors, and ultraviolet detectors.

## 1. Introduction

Semiconducting metal oxides have been intensively developed for optoelectronics and sensor material applications of various transduction platforms in last several decades due their unique material properties of optical transparency based on wide energy bandgap and chemiresistive behavior depending on specific gas adsorption on the surface [1]. Future microelectronics technology utilizing these new functions of metal oxide will therefore lead to a variety of challenging applications and their new consequent industrial market. Among many semiconducting metal oxides, ZnO has been highlighted and extensively investigated in recent years for its distinctive properties and potential applications in optoelectronics, gas sensors, and energy harvesting applications [1,2,3,4,5]. It has a direct band gap of ~3.37 eV at room temperature (RT), a large exciton binding energy of 60 meV, thermal and chemical stability, piezoelectricity, radiation hardness, and the option of wet chemical etching [4,5,6,7].

Numerous methods have been investigated for the synthesis of ZnO crystals thus far, but typical methods adopted for the fabrication of ZnO nanostructures include vapor phase epitaxy, pulsed laser deposition, spray pyrolysis, molecular beam epitaxy, and chemical vapor deposition techniques [3,8,9,10,11]. However, most of these process schemes require complicated facilities and high thermal budget which ultimately hinder low-cost and large scale fabrication on flexible substrates. Among various growth methods for the ZnO nanorods (NRs), a synthesis technique in aqueous solution such as hydrothermal method is very promising because they can proceed at relatively low thermal budget allowing low-cost and large-scale roll to roll fabrication [4,7,12,13]. ZnO also provide a wide variety of nanostructure morphologies such as NRs, nanowires, nano-belts, and nano-flowers for many practical applications [8,9,10,11,12,13]. Fabrication of nano-scale ZnO materials with special morphology and high crystalline quality is of great interest for materials science because of its significance in the scientific research and potential in the miniaturized technological applications.

Drawback of hydrothermal technique is however the presence of huge volume of defects on the surface of grown nanostructures affecting the structural and optical properties of ZnO nanostructures. The optimization of optical and structural properties can be achieved by fully understanding the growth mechanism of nanostructures and how the defects in the crystals behave through the post-surface treatment. However, the morphological control and crystal structure evolution to a low defect-density nanostructures remain challenging to material scientists. In order to suppress the defects in ZnO nanostructures, great amount of research effort has been made by using post growth annealings; for example, Qui et al. post-treated the as-grown ZnO nanowires at 550 °C in different ambient conditions and reported the enhancement of ZnO nanocrystals to the high crystalline quality through vacuum annealing [6], however sufficient understanding how this method contributed to the change in crystalline quality was not achieved.

In this study, we investigate how the post-annealing ambient for the ZnO seed layer (SL), which serves as a platform for the subsequent nanocrystal nucleation, affects the crystalline quality of ZnO NR growth. To develop hydrothermal method to synthesize high crystalline quality ZnO microstructures with a simple post-annealing process at relatively low temperature will be highly desirable due to its easily controllable condition. In addition, the possible defect annihilation mechanism in ZnO NR crystals during the post-annealing is expected to further investigate. If material properties can be precisely tuned by deploying defect chemistry, we can satisfy the precise requirements for applications through an understanding of the defect formation mechanism. For this purpose, we investigated in this study the role of annealing ambient and the evolution of defects in as-annealed SLs as well as ZnO NR crystals by using various surface characterization techniques including X-ray diffraction (XRD), photoluminescence (PL), X-ray photoelectron spectroscopy (XPS), field emission scanning electron microscopy (FE-SEM), Fourier transform infrared (FT-IR) spectroscopy, atomic force microscopy (AFM), and transmission electron microscopy (TEM).

## 2. Experimental Procedure

ZnO NRs were grown on p-Si (100) substrate (2 × 2 cm^2^, 0.01 Ω-cm resistivity of boron doping) using an aqueous solution method in the following way. After the surface cleaning by acetone and isopropyl alcohol sequentially to remove dust and organic contaminants, the substrates were rinsed by de-ionized (DI) water and dried with nitrogen (N_2_) purge followed by moisture removal on hot-plate at 120 °C for 2 min. Aqueous solution route for the growths of ZnO NRs consists of the following two steps. First, a colloidal sol-gel was prepared by mixing zinc acetate dehydrate [Zn(CH_3_COO)_2_·2H_2_O] in an organic solvent of 1-propanol to form a 10 mM concentrate solution. After constantly sonicating the solution for 30 min, it was kept at room temperature longer than 5–6 h for the sol-gel stabilization. This solution for the ZnO SL growth was then spin-coated on the substrate at 3000 rpm for 30 s and baked at 100 °C for 1 min, and the coating was repeated 10 times to achieve a thickness of ~20 nm after annealing. The SL-grown samples were post-annealed at 350 °C in a convection furnace with three different ambients of vacuum (50 mTorr), atmospheric air, and N_2_. Post-annealings for the as-coated SLs were carried out not only to remove the organic residuals and unwanted reaction by-products remaining in the crystals but also to investigate how the crystalline quality of the SL is influenced by the annealing ambient in connection with the material characteristics of the ZnO NRs to be grown afterwards.

**Figure 1:**
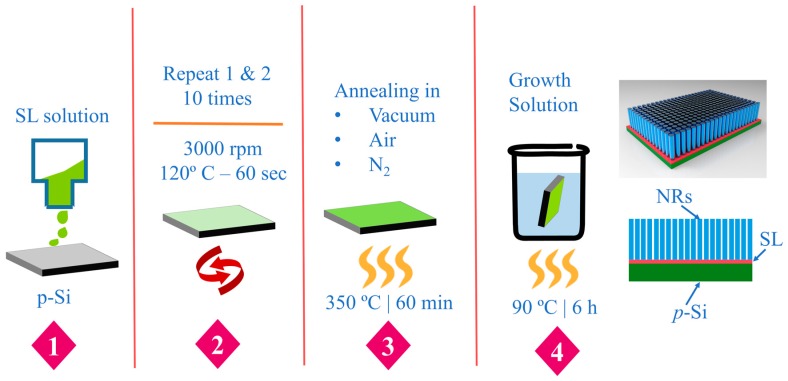
Process flow of ZnO nanorods (NRs) growth with three different post-annealing ambient conditions (vacuum, air and N_2_) for the seed layers (SLs).

In the second step, we immersed the substrates to grow the NR crystals in an equimolar growth solution of (25 mM) zinc nitrate hexahydrate (Zn(NO_3_)_2_∙6H_2_O, 99%) and (25 mM) hexamethylenetetramine (HMT) (C_6_H_12_N_4_, 99.5%) in 250 ml DI water as illustrated in Figure 1. After complete stirring of the growth solution, the seed-grown substrates were placed upside down in a beaker for the NR growth, and the beaker was sealed with an aluminum foil and placed on a hot plate at 90 °C for 5–6 h. Finally, the grown ZnO NRs were washed with DI water and purged with N_2_ gas.

To explore the evolved morphologies and crystalline structures of the as-grown NRs, we performed FE-SEM (Hitachi S-4800S, operated at 15 kV, Suwon, Korea) and cross-sectional TEM (Hitachi 9500 at 300 KV) analysis with selected area electron diffraction (SAED). Crystalline quality and preferred orientation were investigated by XRD (D8 Advance spectrometer of Bruker AXS with Cu Kα 1.540 Å radiation, Seoul, Korea) for each SL and NR samples prepared under different conditions. PL spectroscopy (MFP-3D Bio, Asylum Research, Suwon, Korea) excited by a 325-nm line HeCd laser was also carried out at RT to examine the optical properties of the samples. Chemical bonding and stoichiometric analysis were done by XPS using PHI 5000 Versa Probe (Ulvac-PHI, Suwon, Korea) spectrometer with monochromator Al Kα (1486.6 eV) anode (25.0 W, 15 kV). The bonding configurations of the ZnO SL crystals were investigated by FT-IR spectroscopy (IFS66v/s and Hyperion 3000, Bruker). AFM (N8-NEOS, Bruker) in non-contact mode was also carried out to examine the surface morphologies of the SLs annealed in different ambient conditions.

## 3. Results and Discussion

**Figure 2:**
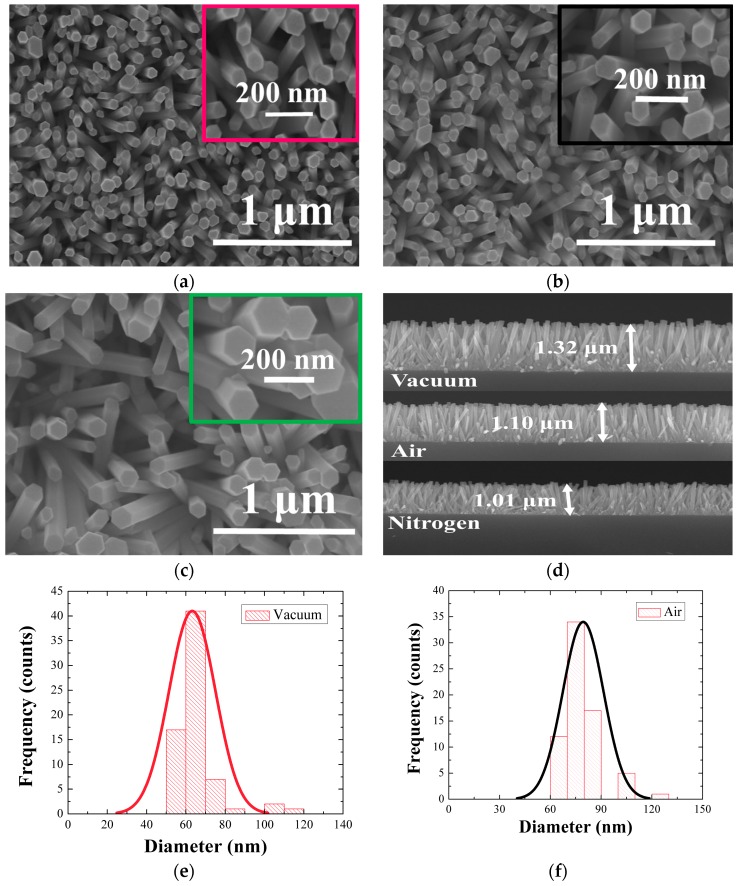

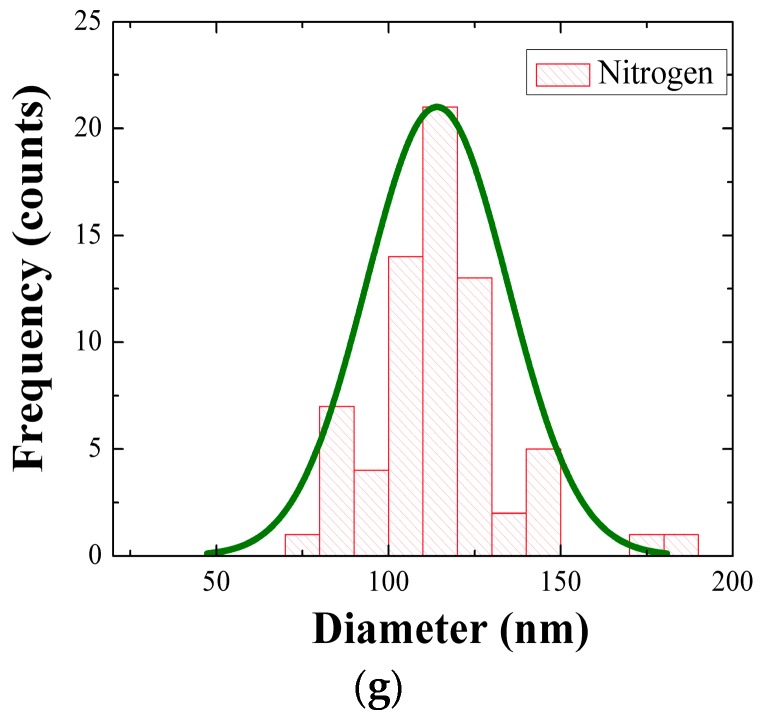
Field emission scanning electron microscopy (FE-SEM) (top view) of the ZnO NRs grown on SLs annealed in (**a**) vacuum; (**b**) air; and (**c**) nitrogen. Cross-sectional views of the ZnO NRs are shown in (**d**). Histogram for the diameter distribution of NRs grown on SLs annealed in (**e**) vacuum; (**f**) air, and (**g**) nitrogen.

The growth morphology of the ZnO NRs was first examined by FE-SEM, and Figure 2a–c show the top views of the NRs grown on SLs annealed under three different ambient conditions of vacuum, air, and N_2_, respectively. Average lengths of the NRs were 1~1.3 μm as shown in the cross-sectional view in Figure 2d, and the NRs grown on the vacuum-annealed SLs exhibited the fastest growth rate. Shown in Figure 2e–g are the histograms of NR diameter distributions, and it was shown that the statistics of NR diameter were also significantly affected by the SL annealing condition. Table 1 summarized the morphology statistics of the NRs grown under three different conditions. The NRs grown on SLs annealed in vacuum showed the smallest mean diameter of 65 nm; on the other hand, the larger diameters of 80 and 115 nm were measured in the cases of annealings in air and N_2_ ambient condition, respectively. Aspect ratio of the NRs was decreased from 20.3 (vacuum) to 13.7 (air) and 8.8 (N_2_) depending on the annealing ambient. The NRs also exhibited the maximum surface density (~240 rods/μm^2^) in the case of vacuum-annealing, but the density was significantly reduced by 33–62% in the cases of different ambient conditions.

**Figure 3:**
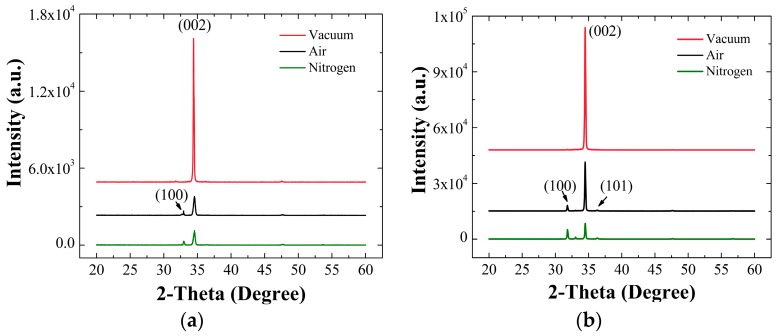
θ-2θ X-ray diffraction (XRD) patterns of (**a**) the SLs annealed in three different ambients; and (**b**) the ZnO NR grown on each different SL.

We obtained well aligned NRs normal to the substrates with pure wurtzite hexagonal faces as shown in each micrograph of Figure 2a–c. The degree of NR c-axis alignment was more prominent in the case of vacuum-annealing than the other cases. Figure 3a,b respectively represent the θ-2θ XRD spectra obtained from the SLs annealed under three different ambient conditions and the NRs grown atop each SL.

From the XRD patterns, it was shown that all reflections were in perfect agreement with the reported indexes of JCPDS files (card number 36-1451) for the hexagonal phase ZnO. In each case, the most intense peaks along (002) orientation were observed from the SLs, and this represents that our ZnO seed crystallites have a preferred orientation along c-axis. Especially, the degree of (002) preferred orientation was the most strong from the SLs annealed in vacuum (at 2θ = 34.44°) with no visible reflection from other planes, while fairly significant (100) reflections were also observed from the SLs when annealed in other ambient conditions [14]. The degree of orientation, *F*(hkl), can be given by the following equation: [15]

(1) $$F\left(\mathrm{hkl}\right)=\frac{\left(P\left(\mathrm{hkl}\right)-\;P_{0}\left(\mathrm{hkl}\right)\right)}{\left(1-\;P_{0}\left(\mathrm{hkl}\right)\right)}$$

where *P*(hkl) = *I*(hkl)/Σ*I*(hkl), *P*_0_(hkl) = *I*_0_(hkl)/Σ(*I*_0_(hkl), *I*(hkl) is the measured peak intensity from (hkl) plane, and *I*_0_(hkl) is the reference peak intensity of (hkl) plane given by JCPDS card No. 36-1451. As summarized in Table 2, the highest value of *F*(002) was obtained from the SLs annealed in vacuum, whereas much lower degree of orientations were measured from the SL crystals annealed in air and N_2_ ambients. The excellent *F*(002) value in the case of vacuum annealing suggests that the gas molecules and/or contaminants present in the annealing ambient can play a very important role in the SL crystal growth and grain coalescence process [14,15,16].

The average grain size d was calculated from full width at half maximum (FWHM) of (002) peak by using Debye-Scherer formula: [15,16]

(2) d=(0.94 λ)/(β Cos θ)

where *λ* is the X-ray wavelength (0.154 nm), β is FWHM in radians, and θ is the Bragg’s diffraction angle. The grain sizes of SL crystals calculated by this method were 55.6 nm (vacuum), 30.4 nm (air), and 28.3 nm (N_2_) in each different annealing ambient condition as shown in Table 2. Annealing in vacuum can promote the grain growth and intergranular coalescence of the SL crystals through the grain boundary diffusion, thereby producing large grain sizes during the post-annealing. However, gas molecules and contaminants in the grain boundaries introduced during the annealing in air or N_2_ ambients can suppress the intergranular diffusion for the evolutionary SL crystal growth [12,15,16].

The XRD patterns for the NRs grown on the SLs annealed under different environment were also shown in Figure 3b. Single strong peak (2θ = 34.47°) along (002) orientation was observed from the NRs grown on the SLs annealed in vacuum, and no other peak linked to any different orientation was found. This reveals that the ZnO NR crystals, in this case, grow dominantly along c-axis in the vertical direction to the substrate. On the other hand, we obtained weak but visible additional peaks from (100) and (101) planes at 31.7° and 36.4°, respectively, with a dominant peak from (002) from the NRs grown on the SLs annealed in air and N_2_. The intensity of (002) peak measured from the vacuum annealing was significantly reduced by 2.5 and 7.6 times in the cases of air and N_2_ ambient annealing, respectively. This depicts that the c-axis alignment of the ZnO NR growth was considerably effected by the annealing ambient condition for the SLs. As was discussed earlier, the SL crystals annealed in vacuum state have a highly preferred orientation toward (002) and large grain sizes. As suggested by many former researchers, each crystalline surface of the SL grain acts as a nuclei for the growth of NRs, and the ZnO NRs tend to dominantly grow along the [001] direction because of its lower surface free energy (1.6 J/m^2^) than those of (100) (3.4 J/m^2^) and (101) (2.0 J/m^2^) planes [17,18]. This can explain why we have a strong c-axis alignment along vertical direction to the substrate when the ZnO NRs grown on the vacuum-annealed SLs.

**Figure 4:**
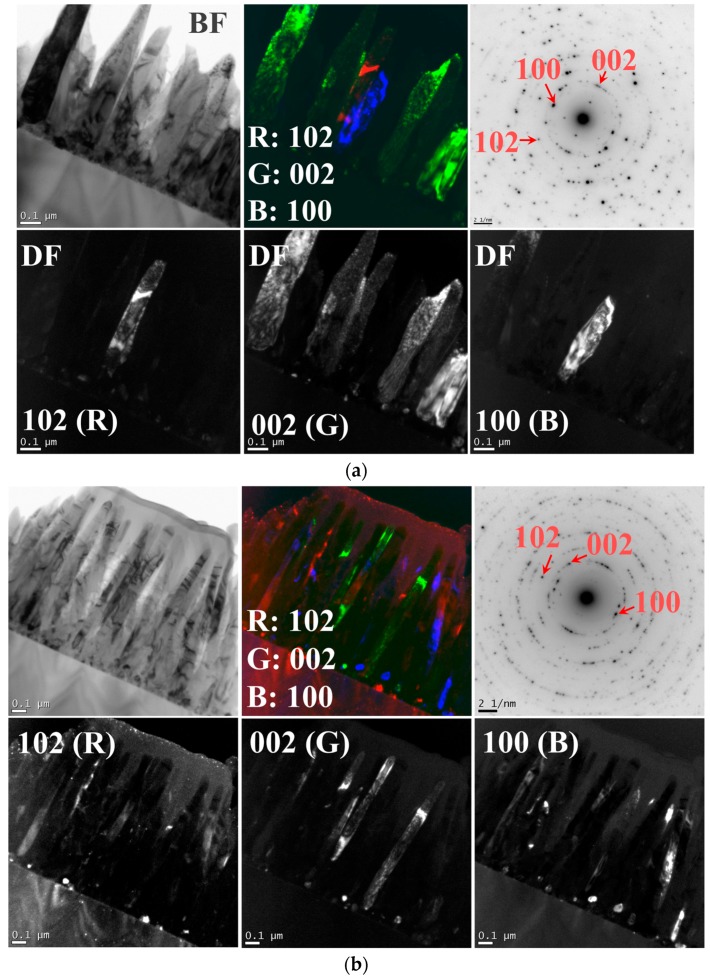

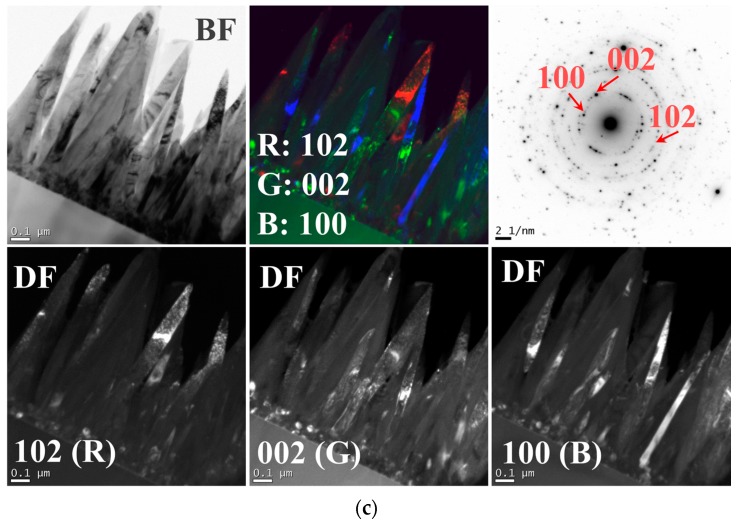
Cross-sectional TEM micrographs of the ZnO NRs grown on SLs annealed in (**a**) vacuum, (**b**) air, and (**c**) nitrogen ambients. Bright-field images and their selected area electron diffraction (SAED) patterns are respectively shown in (top-left) and (top-right). Dark field images are shown according to each different diffraction condition of plane index in (bottom rows) with (top-center) their composite views in three different colors.

TEM dark-field (DF) analyses were carried out for the NR structures grown under three different SL annealing conditions. Figure 4a–c shows the DF images of the NR crystals in the bottom rows, and clear differences in contrast were found from each NR crystal corresponding to their plane indexes diffracted on the SAED patterns. As shown in Figure 4a, most of the NR crystals grown on the vacuum-annealed SLs were bright as observed under (002) diffraction condition, whereas only few NRs showed the bright diffraction contrast under (100) or (102) diffraction condition. The samples prepared under different annealing ambients (such as air or N_2_) showed quite different contrast distribution in NRs depending upon the SL annealing condition as shown in Figure 4b,c. Despite the limitation of TEM analysis in very local areas, this observation was in good agreement with the results of XRD analysis of higher statistical reliability.

**Figure 5:**
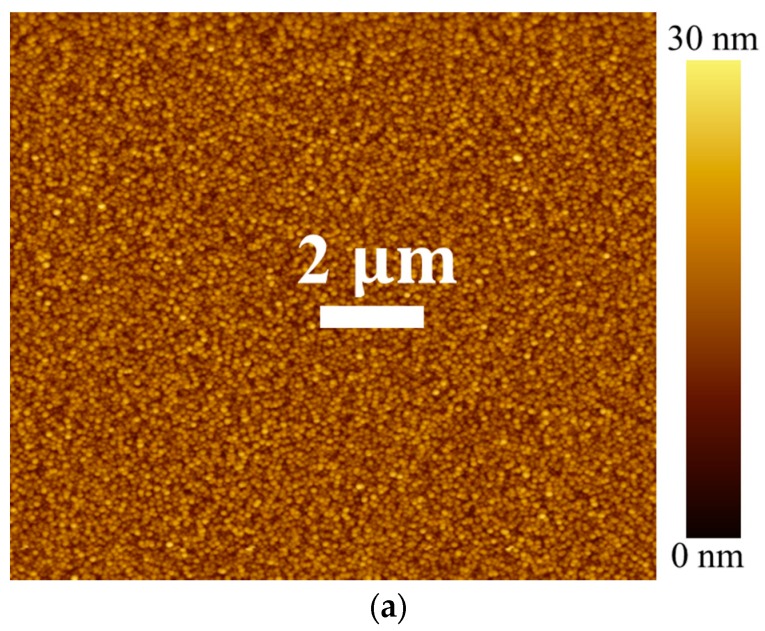

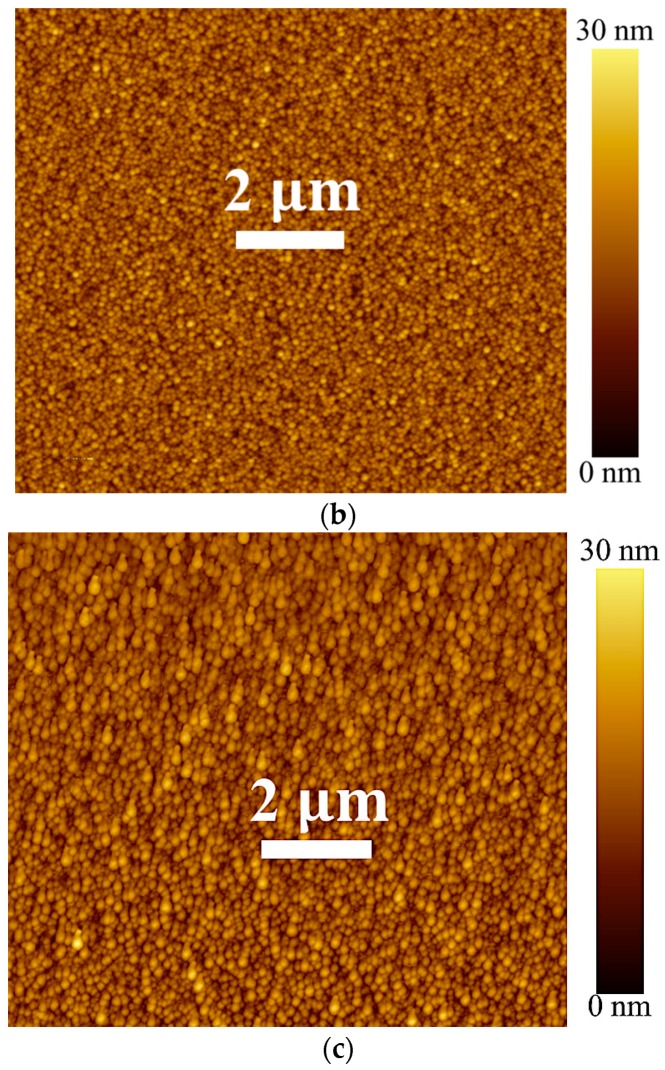
Atomic force microscopy (AFM) images of ZnO SLs annealed in (**a**) vacuum; (**b**) air, and (**c**) Nitrogen ambient.

As shown in Figure 5, we performed AFM for each scanning area (20 × 20 μm^2^) of the SLs annealed in different ambients. The AFM characterizations revealed significantly reduced surface roughness from the SL films annealed in vacuum compared to those annealed in air or N_2_. The root mean square roughness of SL annealed in vacuum was found to be 1.34 nm, while those of SL films annealed in air and N_2_ ambient were 1.84 and 2.05 nm, respectively. This enhancement of surface smoothness from the vacuum-annealed SL crystals can be due to the higher (002) degree of orientation as observed by our XRD analysis [19].

**Figure 6:**
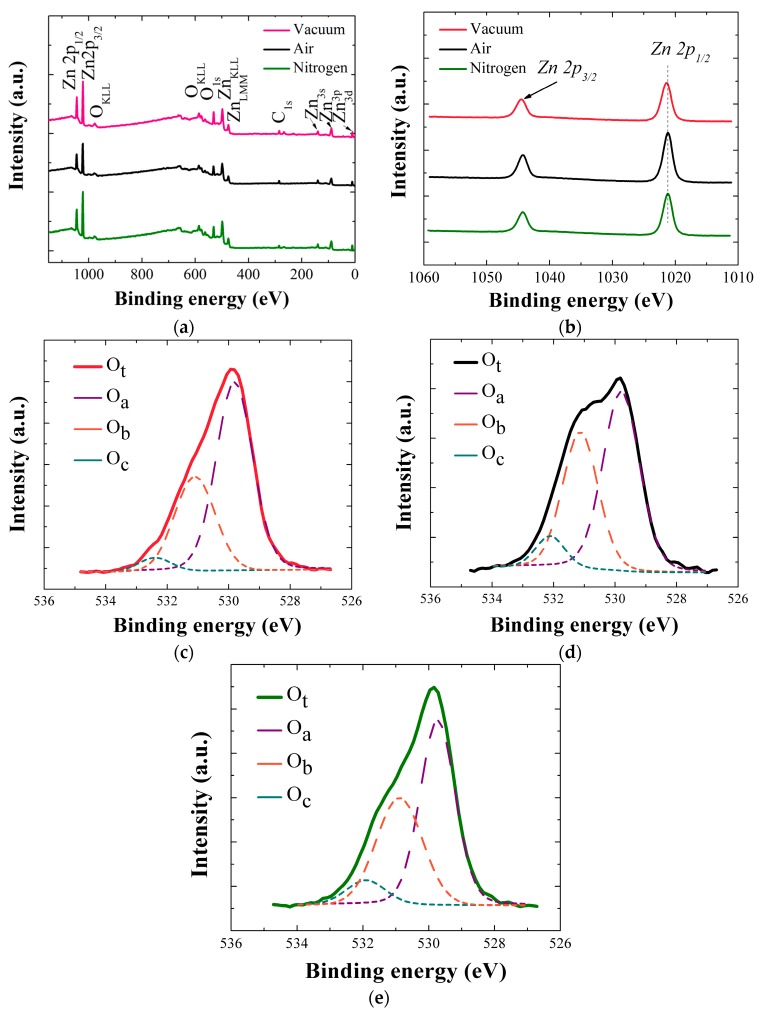
(**a**) Wide scan X-ray photoelectron spectroscopy (XPS) spectra of ZnO SLs annealed in three different ambients; (**b**) High resolution spectra of Zn-2p of the SLs; O1s core level spectra obtained from the ZnO SLs annealed in (**c**) vacuum; (**d**) air, and (**e**) nitrogen ambient are de-convoluted into three distant satellite peaks.

Wide scan XPS spectra for the SLs annealed in different ambient conditions are shown in Figure 6a in a range of 0–1150 eV. Main constituents of Zn and O exhibit various photoelectron peaks of different core-levels and spin-orbital splittings as well as Auger peaks, and some traces of carbon are also detected in the SL crystals. Figure 6b shows two clear core-level Zn peaks of 2p_1/2_ (1044.4 eV) and 2p_3/2_ (1021.3 eV) separated by spin-orbital splitting for each SL crystal annealed in vacuum, air, and N_2_. The peak locations, symmetric shape of the peaks, and spin orbital splitting value (23.1 eV) of the Zn 2p doublet confirms that Zn is present as Zn^2+^ chemical state in ZnO stoichiometry in all cases [16]. However, 2p_1/2_ peak of the SL annealed in vacuum was shifted by 0.17 eV to higher energy, and this shift is attributed to a higher oxidization state of Zn element in the ZnO core matrix.

To examine the change of chemical state in SL ZnO crystals, we observed more closely O1s peaks deconvoluted into three separate satellite peaks of O_a_, O_b_, and O_c_ as shown in Figure 6c–e. O_a_, which is the lowest energy peak at ~530.3 eV, corresponds to the O^2−^ ion in the wurtzite structure of hexagonal ZnO. Because O_a_ is a good measure of stoichiometric oxygen presence in the wurtzite structure, we evaluated the stoichiometry of each ZnO SL crystals by using ∫O_a_/∫Zn in each corresponding annealing condition [16,20,21,22], where ∫O_a_ and ∫Zn respectively represent the peak curve integration of O_a_ and Zn. It is observed in Table 3 that the percentage contribution of O_a_ in O_t_ (O_t_ = O_a_ + O_b_ + O_c_) is the highest in the case of vacuum-annealing. Each percentage value of O_a_, O_b_, and O_c_ in Table 3 was obtained by ∫O_k_/∫O_t_, where ∫O_k_ is the curve integration of individual O1s satellite peak (k = a, b, or c), and ∫O_t_ is the curve integration of total O1s peak. Although ZnO is oxygen-deficient material in nature, but the highest O/Zn atomic ratio of 0.89 was found in the case of vacuum-annealed SL crystals, as shown in Table 3, which is even higher than those of other as-annealed ZnO nanocrystals grown by hydrothermal methods [23]. O_b_ (~531.2 eV) is thought to be originated from O^2–^ ions in the oxygen deficient region within the ZnO matrix [20,21]. The higher binding energy O_c_ component (~532.1 eV) is associated with the presence of loosely bound oxygen such as –CO_3_, –OH species on the surface of ZnO crystals. One interesting observation is that the SL crystals annealed in vacuum show significant reduction in O_c_ contribution compared to those annealed in other ambients as shown in Figure 6c–e and Table 3. This high suppression of O_c_ percentage of the vacuum-annealed samples is most likely due to the effective desorption of O_2_, CO_3_, or OH groups adsorbed on the surface formed during our wet organic solution-based process [23]. As summarized in Table 3, the O_c_ percentage of the vacuum-annealed samples have almost halved in value compared to those of the samples annealed in different ambients.

**Figure 7:**
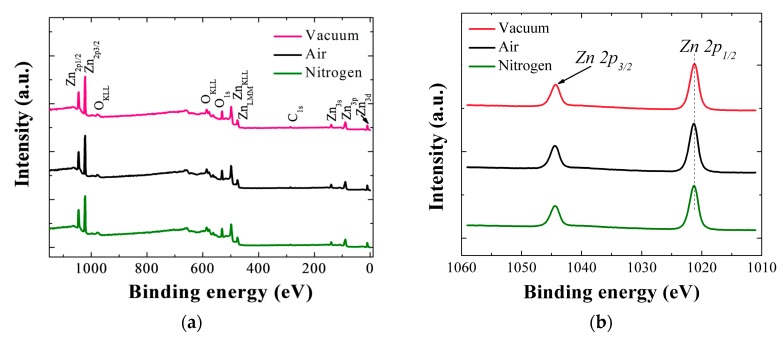

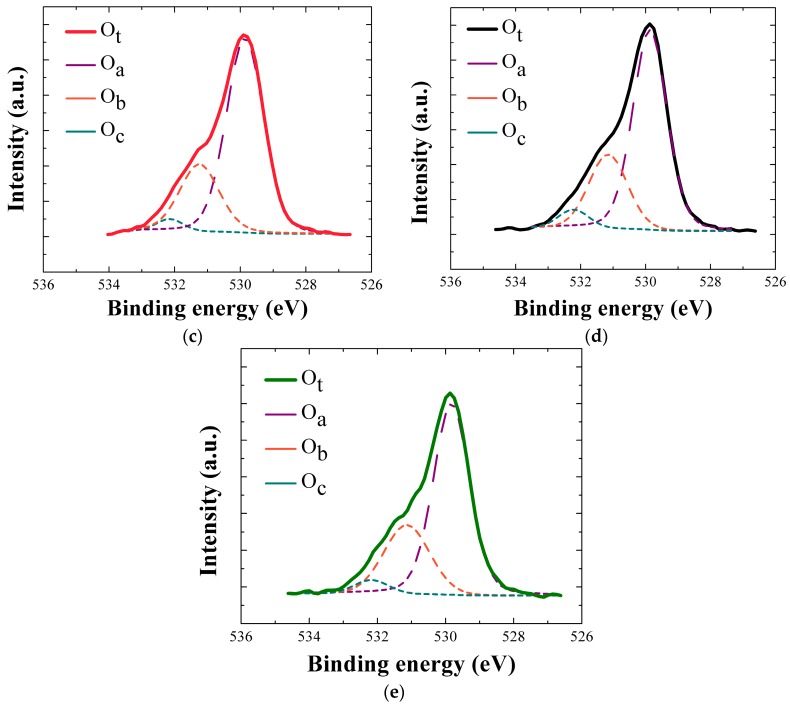
(**a**) Wide scan XPS spectra of ZnO NRs grown on SLs annealed in three different ambients; (**b**) High resolution spectra of Zn-2p of the NRs; O1s core level spectra obtained from the ZnO NRs grown on SLs annealed in (**c**) vacuum; (**d**) air, and (**e**) nitrogen ambient are de-convoluted into three distant satellite peaks.

Shown in Figure 7a,b are the wide scan XPS spectra and Zn 2p core-level patterns obtained from the NRs grown on the SLs annealed in different ambient conditions. Similar trend was shown in the spectra compared to those of the SLs, and all of the peaks can be attributed only to Zn, O, and C elements. Zn 2p doublet has peaks at 1021.3 and 1044.4 eV with spin orbital splitting of 23.1 eV for Zn 2p_1/3_ and Zn 2p_1/2_. From the deconvolution analysis of O1s peaks, as shown in Figure 7c–e, the O/Zn atomic ratio estimated by calculating ∫O_a_/∫Zn was 0.72 for the NR crystals grown on the vacuum-annealed SLs, and this value is 9~11% higher than those of the NRs grown on the SLs in different ambients as shown in the Table 3. We also observed significant reduction of O_c_ percentage in the NR crystals when grown on the SLs annealed in vacuum. From these XPS results, it can be concluded that the reason why the orientation of the ZnO NR crystal is strongly enhanced by the SL vacuum-annealing is most likely based on the following phenomena. Vacuum annealing can remove very effectively the loosely bound oxygen species such as absorbed H_2_O, O_2_, and other oxygenated impurities present on the surface or grain boundaries of the SL crystals formed during the sol-gel based growth. Therefore, through more vigorous grain boundary diffusion process, the SL crystals can grow by merging into larger grains, and at the same time, they can have a more stable (002) preferential orientation in the annealing process. When the NR crystals grow atop the SLs of high crystalline quality with minimized impurities and excellent preferred orientation, they tend to preferentially grow perpendicularly along the c-axis direction, which is the growth process of the lowest activation energy, thereby producing the high crystalline quality of nanostructures.

**Figure 8:**
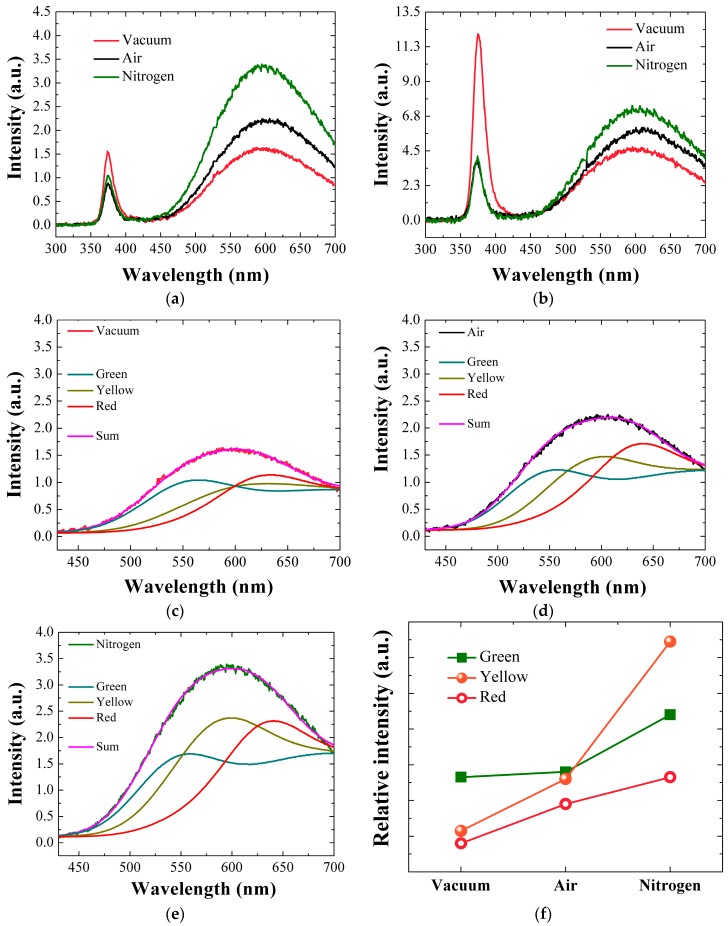
PL spectra for (**a**) the ZnO SLs annealed in three different ambients, and (**b**) the NRs grown on each different SL; De-convoluted visible emission spectra of the SLs annealed in (**c**) vacuum; (**d**) air, and (**e**) nitrogen ambients. Relative intensities of each de-convoluted satellite peak are shown in (**f**).

Figure 8a,b present the RT PL spesctra for the ZnO SLs annealed in different ambients and the NR crystals grown afterwards, and two prominent emission regions were observed in all spectra. The first strong ultra violet (UV) emission in a range of 360–400 nm is due to a near-band-edge emission (NBE) associated with band-to-band excitonic recombination in ZnO, where the highest peak position approximates the band gap of ZnO [4,5,15]. The UV peak position of the SL crystals annealed in vacuum is centered at 374.3 nm (3.312 eV), while the crystals annealed in air and N_2_ show the peaks at 375.2 nm (3.304 eV). A slight red shift of 0.9 nm (8 meV) due to air or N_2_ annealings can be related to the residual stress caused by structural imperfections in the ZnO crystals and the consequent bandgap modulation [15]. The intensity of NBE peak is related to the crystallinity quality of the ZnO SLs [4,14,15,24]. The reason behind this is that the vacuum-annealed seed crystallites exhibit reduction of shallow defects near the band edge and the non-radiative defects are passivated due to annealing effect [25]. On the other hand, annealing in N_2_ or air environment can distort the crystal lattice by creating more defective surface states when the impurities are adsorbed on the surface or grain boundaries of ZnO crystallites [14,26]. The vacuum-annealed SLs showed 33~44% greater UV peak intensity than the samples annealed in air or N_2_ ambient, and this result was in good agreement with our earlier XRD analysis.

Visible light emission ranging from 500 to 700 nm is the second prominent peak as observed in all PL spectra. This emission is known to be originated by deep-level emissions (DLE) caused by the recombination of photo-generated charges with various kinds of intrinsic crystal defects such as neutral or charged O/Zn vacancies, interstitials and anti-sites. The exact nature of each defect state associated with the location of DLE is still a debatable topic; however, the DLE in sol-gel method exists in general due to various kinds of structural defects which are practically difficult to eradicate [4,14,15,24,25,26,27]. As shown in Figure 8a–c, Gaussian-deconvolution analysis was performed on the visible emissions of our PL spectra in order to understand the origin of the DLE. For example, as depicted in Figure 8a, the SL crystals annealed in vacuum exhibit three kinds of DLE, centered at 2.25 eV (green emission), 2.14 eV (yellow emission), and 1.94 eV (red emission). The green emissions is known to be associated with oxygen interstitial (O_int_), oxygen anti-site (O_zn_), and zinc vacancies [25,26,27,28]. Green emission originates from the recombination of electrons from conduction band with holes which are trapped in the deep levels. The yellow emissions are formed in aqueous solution method due to the presence of hydroxyl group. This type of emission is present due to both extrinsic and intrinsic defects. The extrinsic defects like Li or presence of –OH group has been reported, however the intrinsic defect which is the probable cause for yellow emissions is O_int_ [29]. The red emissions are still unknown, and however they can be connected to the transitions related to Zn vacancy complexes. Oxygen and Zn interstitial are also reported to be the primary origins of red emissions [15,26,29].

By changing the annealing environment from air or N_2_ to vacuum, the green emissions were suppressed by 23% or 63% as shown in Figure 8f; therefore, it can be reasonably assumed that many intrinsic defects related to O/Zn vacancies, interstitial, and anti-sites have been reduced in the SL crystals. More significant change was observed in yellow emission, which showed intensity reduction of 39–70% from the vacuum-annealed SLs compared to the emissions in the cases of air and N_2_ annealings. As confirmed by XPS analysis, the contribution of O_c_ percentage decreased most significantly in the case of vacuum annealing, which is closely related to the change in the hydroxyl group in the ZnO crystal. The same trend is observed in the yellow emission which is also closely associated with the hydroxyl group most probably formed during our aqueous solution-based growth process.

Shown in Figure 8b is the PL spectra for the ZnO NRs grown on the SLs annealed under different ambient conditions. In this spectra, more intense UV emissions are observed than from the SLs, which is due to the high crystalline quality of NR crystals, whereas the intensities of UV and visible emission follow the same trend according to the SL annealing ambient conditions. The NR crystals grown on vacuum-annealed SLs produced ~3 times higher emission intensity in UV range than the other samples, which shows a good agreement with our XRD and XPS analysis. Moreover, broadband visible emission from the NR crystals was also effectively suppressed in the case of vacuum annealing for the SLs. Therefore, we can expect greatly reduced intrinsic defects in this NR crystalline state. The seed crystal quality of enhanced c-axis preferential orientation, when annealed in vacuum, can suppress the density of intrinsic defects because most of defect formation in ZnO NRs is deeply related with the distortion of crystalline structure and the interface with underlying seed-crystal grain boundaries.

**Figure 9:**
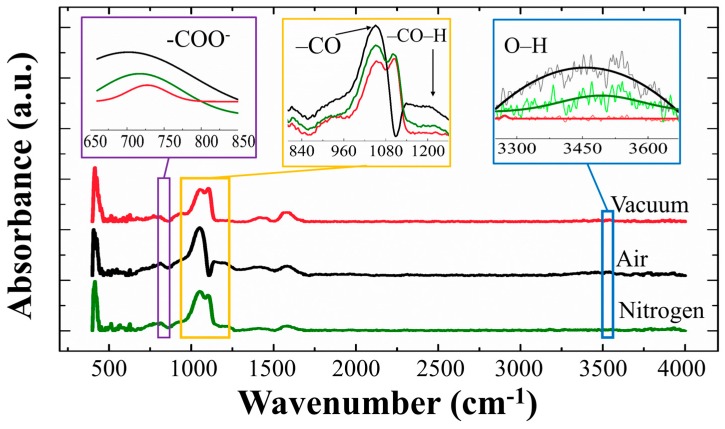
Fourier transform infrared (FT-IR) spectra of ZnO SLs annealed in vacuum, air, and N_2_ ambients. Inset shows an enlarged view of the spectra in a wave number range of 650–850, 800–1250, and 3250–3650 cm^−1^.

The FT-IR characterization for the SLs was carried out in a wave number range of 400–4000 cm^−1^ as shown in Figure 9. A strong absorption in the range 960–1080 cm^−1^ is associated with the stretching vibrations of C–O due zinc acetate dehydrate source [30], and the absorption spectra from 650 to 800 cm^−1^ also corresponds to acetate anion (–COO^−^) twisting and scissoring mode [31]. All of these absorptions from the oxygenated carbons are supposed to be caused by our solution-based SL growth, however they were significantly suppressed in the case of vacuum-annealed SLs [30,31]. A weak absorption from the –CO–H symmetric stretching mode of vibration at ~1230 cm^−1^ was also suppressed in vacuum-annealed SL samples. A broad peak at 3200~3600 cm^−1^ is due to the OH stretch [30,31] and it was shown that this absorption was effectively reduced from the SL crystals when annealed in vacuum or N_2_ ambients of minimized humidity. All the results of this FT-IR analysis are in good agreement with our PL or XPS results, and it can be conclude that the annealing in vacuum is the most effective ambient condition for decontaminating OH and oxygenated carbons in the SL crystals.

## 4. Conclusions

Structural and optical properties of the ZnO NRs grown by hydrothermal method were investigated under different post-annealing ambients for the SLs. Vacuum annealing was the most effective in producing (002) preferred orientation and defect suppression in SL crystals among all annealing conditions. This was verified by XRD analysis by showing a much higher (002) degree of orientation (~0.98) and narrower (002) FWHM (~0.156°) from the SL crystals annealed in vacuum than those annealed in different ambients. The greatest contribution of deconvoluted O_a_ (63.5%) from O1s XPS peak was also observed from the SL crystals annealed in vacuum among all samples annealed under different ambient conditions used in this study. This can be achieved by effectively removing the hydroxyl or oxygenated carbon groups introduced by the aqueous solution-based growth process during the vacuum annealing and by blocking any further impurities introduced from other annealing atmospheres, as investigated by FT-IR spectroscopy and deconvolution analysis of our XPS and PL. ZnO NR crystals grown on the SLs of high crystalline quality and (002) preferred orientation, when annealed in vacuum, also showed excellent degree of (002) orientation with low density of structural and optical defects. As shown in our XRD analysis, 2.5–7.6 times enhanced intensity of (002) peak was obtained from the NR crystals grown on vacuum annealed SLs compared to those grown on SLs annealed in air or N_2_ ambient annealing. The NR crystals grown on vacuum-annealed SLs also produced ~3 times higher PL emission intensity in UV range than the other samples. Research effort on the fabrication of high sensitivity ultraviolet and gas sensors is currently under the work by using the synthesis method of high crystalline quality ZnO NRs examined in this study.

## Figures and Tables

**Table 1:** Morphologies of the ZnO NRs (measured by FE-SEM) prepared under three different SL annealing ambient conditions.

Annealing Ambient for SL	Diameter Range (nm)	Mean Diameter (nm)	Standard Deviation of Diameter (nm)	NR Density (rods/μm^2^)	Aspect Ratio of NR
Vacuum	50–110	65	11.8	~240	20.3
Air	60–130	80	12.0	~160	13.7
Nitrogen	70–190	115	20.4	~90	8.8

**Table 2:** Parameters of the ZnO SL crystals extracted by XRD analysis.

Annealing Ambient for SL	(002) 2θ (°)	(002) FWHM (°)	Grain Size (nm)	(002) Spacing (Å)	Degree of (002) Orientation
Vacuum	34.44	0.156	55.6	2.600	0.98
Air	34.53	0.303	30.4	2.594	0.66
Nitrogen	34.54	0.304	28.3	2.593	0.61

**Table 3:** Percentage values of each O1s satellite peak for the SLs and NRs calculated by dividing the curve integration of individual satellite peak by the curve integration of total peak. O/Zn atomic ratios extracted from each SL and NR crystal are also shown.

Crystal	Annealing Ambient for SL	O_a_ (%)	O_b_ (%)	O_c_ (%)	O/Zn Atomic Ratio
SL	Vacuum	63.5	32.6	3.7	0.89
	Air	54.1	38.8	6.9	0.76
	Nitrogen	53.9	38.3	7.7	0.74
NR	Vacuum	72.3	25.1	2.4	0.72
	Air	68.1	26.9	4.9	0.66
	Nitrogen	67.3	28.6	4.0	0.65
